# 3-Amino­benzonitrile–3,5-dinitro­benzoic acid (1/1)

**DOI:** 10.1107/S1600536811039870

**Published:** 2011-10-05

**Authors:** Xuehua Ding, Shi Wang, Wenrui He, Wei Huang

**Affiliations:** a Jiangsu Key Laboratory of Organic Electronics & Information Displays, Institute of Advanced Materials (IAM), Nanjing University of Posts and Telecommunications, Nanjing 210046, People’s Republic of China

## Abstract

The asymmetric unit of the title co-crystal, C_7_H_6_N_2_·C_7_H_4_N_2_O_6_, contains two formula units of both components. The crystal structure is stabilized by inter­molecular O—H⋯O, N—H⋯O, N—H⋯N and C—H⋯O hydrogen bonds, generating a two-dimensional wave-like network. π–π stacking inter­actions [centroid–centroid distances = 3.702 (2), 3.660 (2)and 3.671 (2) Å] stabilize the crystal packing.

## Related literature

For general background to hydrogen bonding, see: Desiraju (2002 ▶); Prins *et al.* (2001 ▶); Steiner (2002 ▶). For background to the applications of co-crystals, see: Bhatt & Desiraju (2008 ▶); Etter & Baures (1988 ▶); Gao *et al.* (2004 ▶); Hori *et al.* (2009 ▶); Weyna *et al.* (2009 ▶). For the synthesis of co-crystals by complementary functional groups, see: Li *et al.* (2006 ▶); Roy *et al.* (2009 ▶); Wei (2007 ▶).
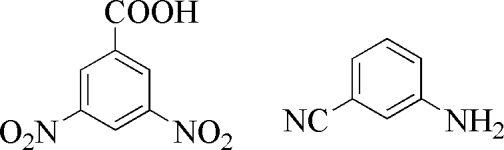


## Experimental

### 

#### Crystal data

C_7_H_6_N_2_·C_7_H_4_N_2_O_6_
*M* *_r_* = 330.26
Triclinic, 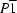

*a* = 7.4547 (15) Å
*b* = 14.260 (3) Å
*c* = 14.845 (3) Å
α = 108.01 (3)°
β = 91.90 (3)°
γ = 93.37 (3)°
*V* = 1496.0 (5) Å^3^
*Z* = 4
Mo *K*α radiation
μ = 0.12 mm^−1^
*T* = 293 K
0.35 × 0.22 × 0.20 mm

#### Data collection

Bruker SMART CCD area-detector diffractometer
Absorption correction: multi-scan (*SADABS*; Sheldrick, 1996 ▶) *T*_min_ = 0.969, *T*_max_ = 0.977
15550 measured reflections
6830 independent reflections
3195 reflections with *I* > 2σ(*I*)
*R*_int_ = 0.056

#### Refinement

*R*[*F* ^2^ > 2σ(*F* ^2^)] = 0.062
*wR*(*F* ^2^) = 0.159
*S* = 0.99
6830 reflections
461 parameters
H atoms treated by a mixture of independent and constrained refinement
Δρ_max_ = 0.16 e Å^−3^
Δρ_min_ = −0.19 e Å^−3^

### 

Data collection: *SMART* (Bruker, 2007 ▶); cell refinement: *SAINT* (Bruker, 2007 ▶); data reduction: *SAINT*; program(s) used to solve structure: *SHELXS97* (Sheldrick, 2008 ▶); program(s) used to refine structure: *SHELXL97* (Sheldrick, 2008 ▶); molecular graphics: *SHELXTL* (Sheldrick, 2008 ▶); software used to prepare material for publication: *SHELXTL*.

## Supplementary Material

Crystal structure: contains datablock(s) I, global. DOI: 10.1107/S1600536811039870/kp2355sup1.cif

Structure factors: contains datablock(s) I. DOI: 10.1107/S1600536811039870/kp2355Isup2.hkl

Supplementary material file. DOI: 10.1107/S1600536811039870/kp2355Isup3.cml

Additional supplementary materials:  crystallographic information; 3D view; checkCIF report

## Figures and Tables

**Table 1:** Hydrogen-bond geometry (Å, °)

*D*—H⋯*A*	*D*—H	H⋯*A*	*D*⋯*A*	*D*—H⋯*A*
O1—H1*A*⋯O2^i^	1.18 (4)	1.41 (4)	2.590 (2)	173 (3)
N6—H6*A*⋯N7^ii^	0.92 (3)	2.32 (4)	3.232 (5)	169 (3)
N6—H6*B*⋯O12^iii^	0.90 (3)	2.57 (3)	2.953 (4)	107 (2)
N8—H8*A*⋯N5^iv^	0.91 (3)	2.37 (3)	3.262 (5)	170 (3)
N8—H8*B*⋯O5^iii^	0.85 (3)	2.48 (4)	3.286 (4)	157 (3)
O7—H9*A*⋯O8^iv^	1.23 (5)	1.38 (5)	2.608 (2)	174 (4)
C5—H5*A*⋯O4^v^	0.93	2.44	3.321 (3)	158
C12—H12*A*⋯O11^ii^	0.93	2.50	3.352 (3)	153
C18—H18*A*⋯O2^vi^	0.96 (3)	2.58 (3)	3.421 (4)	147 (2)
C21—H21*A*⋯O10^vii^	0.93	2.60	3.451 (3)	153

Symmetry codes: (i) 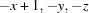; (ii) 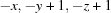; (iii) 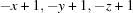; (iv) 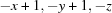; (v) 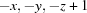; (vi) 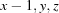; (vii) 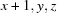.

**Fig. 1.**
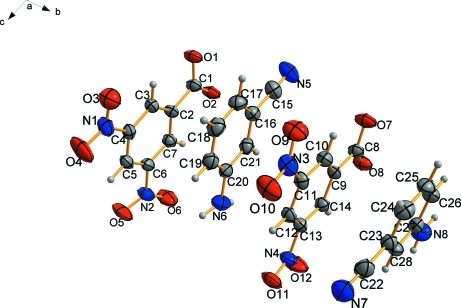
The molecular structure of the title cocrystal with the numbering scheme and 30% probability displacement ellipsoids.

**Fig. 2.**
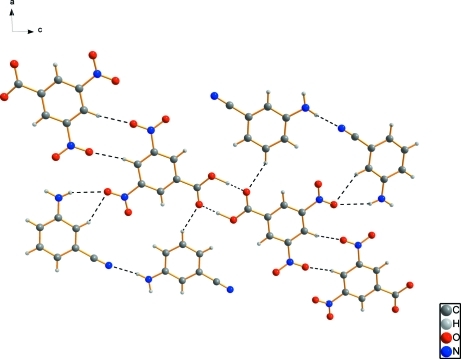
The packing diagram of the molecules viewed along the b axis. Hydrogen bonds are drawn as dashed lines.

**Fig. 3.**
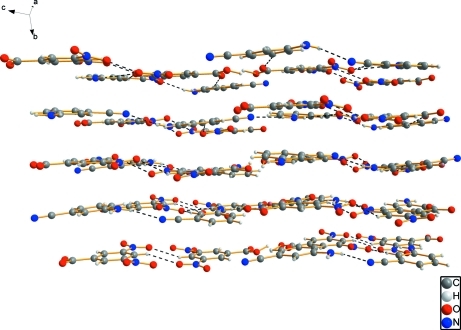
Packing diagram of the molecules. Hydrogen bonds are drawn as dashed lines.

## supplementary crystallographic information

### Comment

The self-assembly of two or more different types of molecules to form a multi-component crystal is greatly fascinating to chemists, known as cocrystals. The considerable effort has been devoted to cocrystal formation over decades, due to its extensive applications in construction of organic solid-state materials, such as in the pharmaceutical industry (Weyna *et al.*, 2009), in organic synthesis (Gao *et al.*, 2004), for promoting crystal growth (Etter & Baures, 1988), as luminescent materials (Hori *et al.*, 2009), and for absolute structure determination (Bhatt & Desiraju, 2008). One of the important ways is the utilisation of self-assembly of small molecules through intermolecular interactions to construct cocrystals, which are one-, two- or three-dimensional networks. In the study of intermolecular interactions the central role is the hydrogen bond. The simple way of preparation of cocrystals is to employ the components containing functional groups with hydrogen bonding capability (Li *et al.*, 2006; Roy *et al.*, 2009; Wei, 2007), such as –COOH and –NH_2_, which can easily result in O—H···N and N—H···O hydrogen bonds.

In this report we have established unambiguously the structure of the cocrystal 3,5-dinitrobenzoic acid with 3-aminobenzonitrile in the solid state by X-ray diffraction analysis. An asymmetric unit of the title compound contains two 3,5-dinitrobenzoic acid and two 3-aminobenzonitrile (Fig. 1). Intermolecular O—H···O, N—H···O, N—H···N and C—H···O hydrogen bonds are observed (Fig. 2, Table 1). The hydrogen bonds  O—H···O between  carboxyl groups results in the dimerization of 3,5-dinitrobenzoic acid. Extensive hydrogen-bonding interactions generate a two-dimensional wave-like network  (Fig. 3).

Additionally, the crystal packing is stabilised by aromatic π–π stacking interactions involving the rings of the asymmetric unit with separation distances between their centroids: *Cg*1(C2→ C7)···*Cg*3 (C16→ C21) of 3.702 (2) Å, *Cg*2(C9→ C14)··· *Cg*3(C16→ C21) of 3.660 (2)Å, and *Cg*2(C9→ C14)···*Cg*4(C23→ C28) of 3.671 (2) Å.

### Experimental

The cocrystals were prepared from stoichiometric amounts of components in water mixed with metanol (in volume ratio 1:1) and left to evaporate slowly at room temperature.

### Refinement

Aromatic H atoms were placed in calculated positions with C—H = 0.93 Å, and refined in riding mode with *U*_iso_(H) = 1.2Ueq(C). H atoms involved in hydrogen bonds were located from differential Fourier maps and refined isotropically.

### Crystal data

**Table:**

C_7_H_6_N_2_·C_7_H_4_N_2_O_6_	*Z* = 4
*M**_r_* = 330.26	*F*(000) = 680
Triclinic, *P*1	*D*_x_ = 1.466 Mg m^−^^3^
Hall symbol: -P 1	Mo *K*α radiation, λ = 0.71073 Å
*a* = 7.4547 (15) Å	Cell parameters from 6830 reflections
*b* = 14.260 (3) Å	θ = 3.0–27.5°
*c* = 14.845 (3) Å	µ = 0.12 mm^−^^1^
α = 108.01 (3)°	*T* = 293 K
β = 91.90 (3)°	Block, colorless
γ = 93.37 (3)°	0.35 × 0.22 × 0.20 mm
*V* = 1496.0 (5) Å^3^	

### Data collection

**Table:**

Bruker SMART CCD area-detector diffractometer	6830 independent reflections
Radiation source: fine-focus sealed tube	3195 reflections with *I* > 2σ(*I*)
graphite	*R*_int_ = 0.056
Detector resolution: 8.366 pixels mm^-1^	θ_max_ = 27.5°, θ_min_ = 3.0°
φ and ω scans	*h* = −9→9
Absorption correction: multi-scan (*SADABS*; Sheldrick, 1996)	*k* = −18→18
*T*_min_ = 0.969, *T*_max_ = 0.977	*l* = −19→19
15550 measured reflections	

### Refinement

**Table:**

Refinement on *F*^2^	Primary atom site location: structure-invariant direct methods
Least-squares matrix: full	Secondary atom site location: difference Fourier map
*R*[*F*^2^ > 2σ(*F*^2^)] = 0.062	Hydrogen site location: inferred from neighbouring sites
*wR*(*F*^2^) = 0.159	H atoms treated by a mixture of independent and constrained refinement
*S* = 0.99	*w* = 1/[σ^2^(*F*_o_^2^) + (0.0591*P*)^2^] where *P* = (*F*_o_^2^ + 2*F*_c_^2^)/3
6830 reflections	(Δ/σ)_max_ < 0.001
461 parameters	Δρ_max_ = 0.16 e Å^−^^3^
0 restraints	Δρ_min_ = −0.19 e Å^−^^3^

### Special details

**Table:**

Geometry. All e.s.d.'s (except the e.s.d. in the dihedral angle between two l.s. planes) are estimated using the full covariance matrix. The cell e.s.d.'s are taken into account individually in the estimation of e.s.d.'s in distances, angles and torsion angles; correlations between e.s.d.'s in cell parameters are only used when they are defined by crystal symmetry. An approximate (isotropic) treatment of cell e.s.d.'s is used for estimating e.s.d.'s involving l.s. planes.
Refinement. Refinement of *F*^2^ against ALL reflections. The weighted *R*-factor *wR* and goodness of fit *S* are based on *F*^2^, conventional *R*-factors *R* are based on *F*, with *F* set to zero for negative *F*^2^. The threshold expression of *F*^2^ > σ(*F*^2^) is used only for calculating *R*-factors(gt) *etc*. and is not relevant to the choice of reflections for refinement. *R*-factors based on *F*^2^ are statistically about twice as large as those based on *F*, and *R*- factors based on ALL data will be even larger.

### Fractional atomic coordinates and isotropic or equivalent isotropic displacement parameters (Å^2^)

**Table:**

	*x*	*y*	*z*	*U*_iso_*/*U*_eq_	
C1	0.4080 (3)	0.01643 (18)	0.11993 (16)	0.0553 (6)	
C2	0.3392 (3)	0.02905 (17)	0.21487 (15)	0.0490 (6)	
C3	0.1637 (3)	−0.00114 (17)	0.22396 (15)	0.0490 (6)	
H3A	0.0862	−0.0291	0.1707	0.059*	
C4	0.1058 (3)	0.01088 (17)	0.31324 (16)	0.0506 (6)	
C5	0.2159 (3)	0.05067 (18)	0.39350 (16)	0.0578 (6)	
H5A	0.1751	0.0569	0.4534	0.069*	
C6	0.3888 (3)	0.08091 (18)	0.38155 (16)	0.0544 (6)	
C7	0.4516 (3)	0.07230 (17)	0.29418 (16)	0.0555 (6)	
H7A	0.5686	0.0953	0.2884	0.067*	
O1	0.3036 (2)	−0.03099 (14)	0.04900 (12)	0.0726 (6)	
H1A	0.363 (5)	−0.035 (3)	−0.025 (3)	0.160 (14)*	
O2	0.5615 (2)	0.05235 (14)	0.11410 (11)	0.0736 (5)	
N1	−0.0818 (3)	−0.01956 (18)	0.32279 (18)	0.0721 (6)	
O3	−0.1815 (2)	−0.04676 (16)	0.25263 (16)	0.0915 (7)	
O4	−0.1288 (3)	−0.0171 (2)	0.40020 (16)	0.1302 (10)	
N2	0.5102 (3)	0.12590 (18)	0.46559 (15)	0.0768 (7)	
O5	0.4669 (3)	0.11480 (19)	0.53961 (14)	0.1112 (8)	
O6	0.6490 (3)	0.16961 (19)	0.45609 (15)	0.1188 (9)	
C8	0.3501 (3)	0.50842 (18)	0.10190 (17)	0.0544 (6)	
C9	0.2362 (3)	0.50677 (16)	0.18160 (15)	0.0488 (6)	
C10	0.0604 (3)	0.46720 (17)	0.16271 (16)	0.0536 (6)	
H10A	0.0117	0.4431	0.1007	0.064*	
C11	−0.0412 (3)	0.46423 (18)	0.23756 (17)	0.0527 (6)	
C12	0.0242 (3)	0.49980 (17)	0.33016 (16)	0.0544 (6)	
H12A	−0.0470	0.4979	0.3800	0.065*	
C13	0.1991 (3)	0.53816 (17)	0.34579 (15)	0.0527 (6)	
C14	0.3074 (3)	0.54240 (17)	0.27369 (16)	0.0517 (6)	
H14A	0.4260	0.5687	0.2868	0.062*	
N3	−0.2260 (3)	0.41929 (18)	0.21744 (18)	0.0714 (6)	
O7	0.2803 (2)	0.47150 (15)	0.01875 (13)	0.0800 (6)	
H9A	0.379 (6)	0.469 (3)	−0.047 (3)	0.203 (18)*	
O8	0.5087 (2)	0.54478 (14)	0.12114 (12)	0.0714 (5)	
O9	−0.2761 (2)	0.37659 (17)	0.13605 (16)	0.0941 (7)	
O10	−0.3199 (2)	0.42753 (18)	0.28450 (15)	0.1015 (7)	
O11	0.1742 (3)	0.58417 (17)	0.50753 (13)	0.1032 (7)	
O12	0.4324 (3)	0.6007 (2)	0.45542 (14)	0.1205 (9)	
N4	0.2750 (3)	0.57692 (17)	0.44339 (15)	0.0744 (6)	
N5	0.3740 (4)	0.2057 (2)	−0.0240 (2)	0.1150 (10)	
C15	0.2997 (4)	0.2144 (2)	0.0436 (2)	0.0796 (9)	
C16	0.2076 (3)	0.22522 (19)	0.12900 (18)	0.0599 (7)	
C17	0.0298 (4)	0.1905 (2)	0.1228 (2)	0.0735 (8)	
H17A	−0.0302	0.1603	0.0641	0.088*	
C18	−0.0566 (4)	0.2014 (2)	0.2050 (2)	0.0765 (8)	
H18A	−0.180 (4)	0.1780 (19)	0.2036 (17)	0.086 (9)*	
C19	0.0298 (4)	0.24544 (19)	0.2909 (2)	0.0685 (7)	
H19A	−0.0323	0.2521	0.3456	0.082*	
C20	0.2079 (3)	0.28052 (17)	0.29893 (18)	0.0596 (7)	
C21	0.2961 (3)	0.27005 (18)	0.21579 (18)	0.0610 (7)	
H21A	0.4159	0.2936	0.2189	0.073*	
N6	0.2934 (5)	0.3273 (2)	0.3859 (2)	0.0891 (8)	
H6A	0.240 (5)	0.317 (3)	0.437 (3)	0.127 (14)*	
H6B	0.410 (4)	0.341 (2)	0.378 (2)	0.109 (13)*	
N7	−0.1478 (4)	0.7302 (2)	0.4340 (2)	0.1190 (11)	
C22	−0.0799 (4)	0.7346 (2)	0.3675 (2)	0.0822 (9)	
C23	0.0139 (3)	0.73855 (18)	0.28564 (19)	0.0606 (7)	
C24	−0.0753 (4)	0.7084 (2)	0.1977 (2)	0.0732 (8)	
H24A	−0.1966	0.6869	0.1906	0.088*	
C25	0.0187 (4)	0.7110 (2)	0.1210 (2)	0.0771 (8)	
H25A	−0.0403	0.6921	0.0613	0.093*	
C26	0.1980 (4)	0.74096 (19)	0.13031 (18)	0.0664 (7)	
H26A	0.2596	0.7403	0.0767	0.080*	
C27	0.2891 (3)	0.77223 (18)	0.21843 (18)	0.0584 (6)	
C28	0.1939 (3)	0.76999 (17)	0.29630 (17)	0.0588 (6)	
H28A	0.2519	0.7899	0.3563	0.071*	
N8	0.4678 (4)	0.8027 (2)	0.2284 (3)	0.0883 (8)	
H8A	0.524 (4)	0.806 (2)	0.176 (2)	0.103 (12)*	
H8B	0.517 (5)	0.825 (3)	0.284 (3)	0.126 (15)*	

### Atomic displacement parameters (Å^2^)

**Table:**

	*U*^11^	*U*^22^	*U*^33^	*U*^12^	*U*^13^	*U*^23^
C1	0.0514 (14)	0.0662 (17)	0.0432 (14)	−0.0022 (12)	0.0060 (12)	0.0104 (13)
C2	0.0527 (14)	0.0535 (15)	0.0398 (13)	0.0060 (11)	0.0078 (11)	0.0119 (11)
C3	0.0552 (14)	0.0509 (14)	0.0424 (13)	0.0036 (11)	0.0047 (10)	0.0165 (11)
C4	0.0527 (13)	0.0536 (15)	0.0512 (15)	0.0053 (11)	0.0119 (11)	0.0233 (12)
C5	0.0707 (17)	0.0647 (17)	0.0426 (14)	0.0115 (13)	0.0127 (12)	0.0215 (13)
C6	0.0657 (15)	0.0574 (16)	0.0389 (13)	0.0058 (12)	0.0027 (11)	0.0129 (12)
C7	0.0528 (14)	0.0614 (16)	0.0509 (15)	0.0007 (12)	0.0048 (12)	0.0158 (13)
O1	0.0636 (10)	0.1044 (15)	0.0372 (10)	−0.0161 (10)	0.0058 (8)	0.0072 (10)
O2	0.0595 (11)	0.1067 (15)	0.0433 (10)	−0.0163 (10)	0.0104 (8)	0.0101 (10)
N1	0.0651 (15)	0.0872 (17)	0.0704 (17)	−0.0002 (12)	0.0185 (13)	0.0335 (14)
O3	0.0583 (11)	0.1231 (18)	0.0878 (15)	−0.0115 (11)	0.0058 (11)	0.0283 (14)
O4	0.0954 (16)	0.225 (3)	0.0855 (16)	−0.0204 (16)	0.0314 (13)	0.0746 (18)
N2	0.0839 (17)	0.0953 (19)	0.0439 (14)	−0.0009 (14)	−0.0024 (12)	0.0128 (13)
O5	0.1111 (17)	0.174 (2)	0.0451 (12)	−0.0154 (15)	−0.0068 (11)	0.0355 (14)
O6	0.1040 (17)	0.166 (2)	0.0704 (14)	−0.0510 (17)	−0.0168 (12)	0.0260 (15)
C8	0.0540 (15)	0.0601 (16)	0.0460 (15)	0.0010 (12)	0.0094 (12)	0.0121 (12)
C9	0.0539 (14)	0.0493 (14)	0.0433 (13)	0.0032 (11)	0.0108 (11)	0.0140 (11)
C10	0.0548 (14)	0.0584 (16)	0.0475 (14)	0.0034 (12)	0.0030 (11)	0.0163 (12)
C11	0.0449 (13)	0.0606 (16)	0.0584 (16)	0.0046 (11)	0.0109 (11)	0.0260 (13)
C12	0.0607 (15)	0.0575 (16)	0.0506 (15)	0.0067 (12)	0.0184 (12)	0.0231 (13)
C13	0.0616 (15)	0.0559 (15)	0.0408 (14)	−0.0003 (12)	0.0070 (11)	0.0157 (12)
C14	0.0508 (13)	0.0543 (15)	0.0487 (14)	−0.0002 (11)	0.0077 (11)	0.0143 (12)
N3	0.0505 (13)	0.0916 (18)	0.0756 (17)	0.0002 (12)	0.0066 (13)	0.0320 (15)
O7	0.0728 (12)	0.1110 (16)	0.0450 (11)	−0.0172 (10)	0.0127 (9)	0.0115 (11)
O8	0.0577 (11)	0.0959 (14)	0.0535 (11)	−0.0099 (10)	0.0156 (8)	0.0143 (10)
O9	0.0639 (12)	0.1283 (19)	0.0836 (15)	−0.0143 (11)	−0.0095 (11)	0.0288 (14)
O10	0.0590 (12)	0.158 (2)	0.0921 (16)	−0.0105 (12)	0.0214 (11)	0.0475 (15)
O11	0.1285 (17)	0.132 (2)	0.0450 (11)	−0.0182 (14)	0.0226 (12)	0.0245 (12)
O12	0.0902 (15)	0.187 (3)	0.0614 (13)	−0.0423 (16)	−0.0114 (11)	0.0159 (14)
N4	0.0881 (17)	0.0858 (18)	0.0440 (13)	−0.0099 (14)	0.0041 (13)	0.0154 (12)
N5	0.121 (2)	0.157 (3)	0.080 (2)	0.017 (2)	0.0235 (18)	0.053 (2)
C15	0.085 (2)	0.091 (2)	0.070 (2)	0.0088 (17)	0.0048 (17)	0.0367 (19)
C16	0.0650 (16)	0.0612 (17)	0.0546 (16)	0.0017 (13)	0.0008 (13)	0.0205 (14)
C17	0.0744 (18)	0.0696 (19)	0.0684 (19)	−0.0075 (15)	−0.0126 (15)	0.0137 (15)
C18	0.0634 (18)	0.075 (2)	0.087 (2)	−0.0125 (15)	0.0020 (17)	0.0222 (18)
C19	0.0726 (18)	0.0608 (18)	0.0721 (19)	0.0004 (14)	0.0124 (15)	0.0207 (15)
C20	0.0746 (17)	0.0462 (15)	0.0561 (17)	0.0016 (13)	−0.0079 (13)	0.0148 (13)
C21	0.0554 (14)	0.0606 (17)	0.0694 (18)	−0.0029 (12)	−0.0033 (13)	0.0258 (14)
N6	0.107 (2)	0.093 (2)	0.0613 (18)	−0.0088 (18)	−0.0128 (17)	0.0202 (15)
N7	0.146 (3)	0.112 (3)	0.107 (2)	0.0211 (19)	0.064 (2)	0.039 (2)
C22	0.091 (2)	0.071 (2)	0.087 (2)	0.0170 (16)	0.0310 (18)	0.0229 (18)
C23	0.0673 (16)	0.0535 (16)	0.0616 (17)	0.0104 (13)	0.0117 (14)	0.0168 (13)
C24	0.0606 (16)	0.0712 (19)	0.083 (2)	0.0050 (14)	−0.0036 (15)	0.0177 (17)
C25	0.090 (2)	0.076 (2)	0.0602 (18)	0.0097 (16)	−0.0166 (16)	0.0141 (16)
C26	0.0819 (19)	0.0663 (18)	0.0503 (16)	0.0071 (15)	0.0052 (14)	0.0169 (14)
C27	0.0653 (16)	0.0498 (15)	0.0593 (17)	0.0047 (12)	0.0038 (13)	0.0156 (13)
C28	0.0711 (17)	0.0541 (16)	0.0477 (15)	0.0046 (13)	−0.0056 (12)	0.0117 (13)
N8	0.0720 (18)	0.102 (2)	0.084 (2)	−0.0103 (15)	0.0072 (17)	0.0221 (19)

### Geometric parameters (Å, °)

**Table:**

C1—O2	1.243 (3)	N3—O10	1.216 (3)
C1—O1	1.271 (3)	O7—H9A	1.23 (5)
C1—C2	1.478 (3)	O11—N4	1.216 (3)
C2—C7	1.376 (3)	O12—N4	1.194 (3)
C2—C3	1.377 (3)	N5—C15	1.139 (3)
C3—C4	1.371 (3)	C15—C16	1.433 (4)
C3—H3A	0.9300	C16—C21	1.374 (3)
C4—C5	1.370 (3)	C16—C17	1.377 (3)
C4—N1	1.464 (3)	C17—C18	1.370 (4)
C5—C6	1.369 (3)	C17—H17A	0.9300
C5—H5A	0.9300	C18—C19	1.356 (4)
C6—C7	1.366 (3)	C18—H18A	0.96 (3)
C6—N2	1.466 (3)	C19—C20	1.379 (3)
C7—H7A	0.9300	C19—H19A	0.9300
O1—H1A	1.18 (4)	C20—N6	1.371 (3)
N1—O4	1.203 (3)	C20—C21	1.389 (3)
N1—O3	1.206 (3)	C21—H21A	0.9300
N2—O5	1.209 (3)	N6—H6A	0.92 (3)
N2—O6	1.211 (3)	N6—H6B	0.90 (3)
C8—O8	1.250 (3)	N7—C22	1.140 (3)
C8—O7	1.263 (3)	C22—C23	1.437 (4)
C8—C9	1.484 (3)	C23—C24	1.376 (4)
C9—C14	1.378 (3)	C23—C28	1.377 (3)
C9—C10	1.380 (3)	C24—C25	1.365 (4)
C10—C11	1.375 (3)	C24—H24A	0.9300
C10—H10A	0.9300	C25—C26	1.369 (4)
C11—C12	1.371 (3)	C25—H25A	0.9300
C11—N3	1.466 (3)	C26—C27	1.384 (3)
C12—C13	1.368 (3)	C26—H26A	0.9300
C12—H12A	0.9300	C27—N8	1.365 (3)
C13—C14	1.376 (3)	C27—C28	1.384 (3)
C13—N4	1.462 (3)	C28—H28A	0.9300
C14—H14A	0.9300	N8—H8A	0.91 (3)
N3—O9	1.208 (3)	N8—H8B	0.85 (3)
			
O2—C1—O1	124.3 (2)	O9—N3—C11	118.6 (2)
O2—C1—C2	118.9 (2)	O10—N3—C11	117.5 (2)
O1—C1—C2	116.8 (2)	C8—O7—H9A	116.9 (18)
C7—C2—C3	120.3 (2)	O12—N4—O11	123.8 (2)
C7—C2—C1	119.4 (2)	O12—N4—C13	118.0 (2)
C3—C2—C1	120.3 (2)	O11—N4—C13	118.3 (2)
C4—C3—C2	118.5 (2)	N5—C15—C16	179.5 (3)
C4—C3—H3A	120.8	C21—C16—C17	120.7 (2)
C2—C3—H3A	120.8	C21—C16—C15	120.2 (2)
C5—C4—C3	122.7 (2)	C17—C16—C15	119.1 (3)
C5—C4—N1	119.0 (2)	C18—C17—C16	118.5 (3)
C3—C4—N1	118.4 (2)	C18—C17—H17A	120.7
C6—C5—C4	117.1 (2)	C16—C17—H17A	120.7
C6—C5—H5A	121.5	C19—C18—C17	121.1 (3)
C4—C5—H5A	121.5	C19—C18—H18A	118.0 (15)
C7—C6—C5	122.4 (2)	C17—C18—H18A	120.9 (15)
C7—C6—N2	118.6 (2)	C18—C19—C20	121.4 (3)
C5—C6—N2	118.9 (2)	C18—C19—H19A	119.3
C6—C7—C2	119.0 (2)	C20—C19—H19A	119.3
C6—C7—H7A	120.5	N6—C20—C19	121.1 (3)
C2—C7—H7A	120.5	N6—C20—C21	121.1 (3)
C1—O1—H1A	113.5 (16)	C19—C20—C21	117.7 (2)
O4—N1—O3	123.0 (2)	C16—C21—C20	120.5 (2)
O4—N1—C4	118.5 (3)	C16—C21—H21A	119.7
O3—N1—C4	118.5 (2)	C20—C21—H21A	119.7
O5—N2—O6	124.3 (2)	C20—N6—H6A	116 (2)
O5—N2—C6	117.6 (2)	C20—N6—H6B	109 (2)
O6—N2—C6	118.1 (2)	H6A—N6—H6B	129 (3)
O8—C8—O7	124.3 (2)	N7—C22—C23	177.1 (4)
O8—C8—C9	118.2 (2)	C24—C23—C28	121.0 (2)
O7—C8—C9	117.5 (2)	C24—C23—C22	119.8 (3)
C14—C9—C10	120.4 (2)	C28—C23—C22	119.2 (3)
C14—C9—C8	120.0 (2)	C25—C24—C23	118.4 (3)
C10—C9—C8	119.6 (2)	C25—C24—H24A	120.8
C11—C10—C9	118.6 (2)	C23—C24—H24A	120.8
C11—C10—H10A	120.7	C24—C25—C26	121.3 (3)
C9—C10—H10A	120.7	C24—C25—H25A	119.3
C12—C11—C10	122.8 (2)	C26—C25—H25A	119.3
C12—C11—N3	118.6 (2)	C25—C26—C27	120.9 (3)
C10—C11—N3	118.6 (2)	C25—C26—H26A	119.5
C13—C12—C11	116.8 (2)	C27—C26—H26A	119.5
C13—C12—H12A	121.6	N8—C27—C26	121.3 (3)
C11—C12—H12A	121.6	N8—C27—C28	120.8 (3)
C12—C13—C14	122.9 (2)	C26—C27—C28	117.8 (2)
C12—C13—N4	118.8 (2)	C23—C28—C27	120.6 (2)
C14—C13—N4	118.2 (2)	C23—C28—H28A	119.7
C13—C14—C9	118.5 (2)	C27—C28—H28A	119.7
C13—C14—H14A	120.7	C27—N8—H8A	118 (2)
C9—C14—H14A	120.7	C27—N8—H8B	118 (2)
O9—N3—O10	123.9 (2)	H8A—N8—H8B	123 (3)

### Hydrogen-bond geometry (Å, °)

**Table:**

*D*—H···*A*	*D*—H	H···*A*	*D*···*A*	*D*—H···*A*
O1—H1A···O2^i^	1.18 (4)	1.41 (4)	2.590 (2)	173 (3)
N6—H6A···N7^ii^	0.92 (3)	2.32 (4)	3.232 (5)	169 (3)
N6—H6B···O12^iii^	0.90 (3)	2.57 (3)	2.953 (4)	107 (2)
N8—H8A···N5^iv^	0.91 (3)	2.37 (3)	3.262 (5)	170 (3)
N8—H8B···O5^iii^	0.85 (3)	2.48 (4)	3.286 (4)	157 (3)
O7—H9A···O8^iv^	1.23 (5)	1.38 (5)	2.608 (2)	174 (4)
C5—H5A···O4^v^	0.93	2.44	3.321 (3)	158.
C12—H12A···O11^ii^	0.93	2.50	3.352 (3)	153.
C18—H18A···O2^vi^	0.96 (3)	2.58 (3)	3.421 (4)	147 (2)
C21—H21A···O10^vii^	0.93	2.60	3.451 (3)	153.

Symmetry codes: (i) −*x*+1, −*y*, −*z*; (ii) −*x*, −*y*+1, −*z*+1; (iii) −*x*+1, −*y*+1, −*z*+1; (iv) −*x*+1, −*y*+1, −*z*; (v) −*x*, −*y*, −*z*+1; (vi) *x*−1, *y*, *z*; (vii) *x*+1, *y*, *z*.
